# Cyclic Peptide Extracts Derived From *Pseudostellaria heterophylla* Ameliorates COPD *via* Regulation of the TLR4/MyD88 Pathway Proteins

**DOI:** 10.3389/fphar.2020.00850

**Published:** 2020-06-09

**Authors:** Feng Lu, Han Yang, Si-ding Lin, Li Zhao, Chang Jiang, Zhi-bin Chen, Ying-ying Liu, Yong-jun Kan, Juan Hu, Wen-sheng Pang

**Affiliations:** ^1^School of Pharmacy, Fujian University of Traditional Chinese Medicine, Fuzhou, China; ^2^Respiratory Department, The Second Affiliated Hospital of Fujian Traditional Chinese Medical University, Fuzhou, China; ^3^Institute of Materia Medica, Fujian Academy of Traditional Chinese Medicine, Fuzhou, China

**Keywords:** *Pseudostellaria heterophylla*, cyclic peptide, monomer structure, TLR4-MyD88-JNK/p38 pathway, anti-chronic obstructive pulmonary disease

## Abstract

We have explored the method of extraction and purification of cyclic-peptide extract (CPE) from *Pseudostellaria heterophylla* (Miq.) Pax. (Taizishen, TZS), characterized the structure about cyclic-peptide compounds and investigated the biological activity of CPE attenuating chronic obstructive pulmonary disease (COPD) in rats. The CPE from TZS was obtained by ethyl acetate, petroleum ether, hot water extraction, and alcohol-precipitation. Cyclic-peptide structures were distinguished using ultra-high performance liquid chromatography-quadrupole time-of-flight tandem mass spectrometry (UPLC-Q-TOF-MS/MS). Rats were induced by solid combustibles smoke (SCS) for the COPD model, and the anti-COPD activity of CPE was detected using lung airway resistance and dynamic lung compliance, as well as pulmonary tissue hematoxylin and eosin (HE) staining. The relevant inflammatory cytokines were assayed by enzyme-linked immunosorbent assay (ELISA). CPE obtained from TZS contained 12 cyclic-peptide constituents; the purity was up to 92.94%. CPE (200, 400, or 500 mg/kg/day) was given to SCS-induced COPD model rats orally for 15 days. The results showed that in rats given CPE (400 mg/kg/day) there was a sharp fall in lung airway resistance but a rise in dynamic lung compliance. The image analysis of lung tissue sections suggested that CPE could decrease the degree of alveolar destruction (*p <*0.05), alleviate lung inflammation, increase alveolar space, and improve the infiltration of inflammatory cells. CPE was found to reduce the levels of TNF-α, but increase IL-10, adjusting multiple cytokines in rat serum; the TLR4 mRNA, MyD88 mRNA and AP-1 mRNA levels, the expressing levels of p-JNK, p-p38 and p-TAK1 protein were significantly down regulated in rat alveolar macrophages. CPE intervention could improve the pulmonary ventilation function on COPD rats, which may be related to its effect in inhibiting the abnormal activation of the TLR4-MyD88-JNK/p38 pathway. This is the first report that the CPE of TZS lessens the severity of COPD episodes. The new preparation process of CPEs implements the anticipated goal, which is to refine CPE and actualize quality control.

## Introduction

Chronic obstructive pulmonary disease (COPD) is a noteworthy public health problem due to its morbidity and mortality (Lindberg et al., 2012). COPD exhibits symptoms of airflow blockage and breathlessness and includes emphysema, chronic bronchitis, or even asthma. Diagnosis of COPD can be best addressed by a combination of morphology and function. The morphological imaging of the lung parenchyma and airways can be detected using the three-dimensional high-resolution computed tomography (CT) technique. The image information obtained by functional imaging is perfusion, lung mechanics, and ventilation by magnetic resonance imaging (MRI). The comprehensive diagnostics of CT complemented with MRI are able to achieve a more sensitive detection (Ley et al., 2008). The development of COPD is mainly attributable to smoking, but genetic factors and respiratory infections are some of the most common causes of asthma; air pollution can aggravate asthma. In developing countries, air quality is thought to have a larger role in the progression of COPD (David and Victor, 2006).

COPD is closely correlated with a chronic inflammatory response, which is characterized by an increased amount of macrophages, neutrophils, and T lymphocytes in the small airways and lung parenchyma (Pelaia et al., 2006). In contrast to asthma, the inflammatory mediators involved in COPD are not clear, but inflammation comprises many mediators, factors, and pathways such as inflammatory peptides, lipid mediators, active oxygen, and cytokines that cause alveolar destruction and small airway fibrosis. Recognizing the inflammatory factors and understanding the interaction mechanism is crucial for anti-inflammatory treatments of COPD (Barnes et al., 2004).

COPD is a non-specific inflammatory disease, and an abnormal immune response plays an important role. Toll-like receptors (TLRs) have been studied that are a cornerstone of the innate immune system and play a pluripotent role in stable COPD. Myeloid differentiation primary response protein 88 (MyD88) is a critical adaptor protein involved in the TLR family signaling pathway and regulates immune responses and inflammation. Targeting MyD88 is one way to treat COPD (Di Stefano et al., 2017; Franco et al., 2018).

Coughing and wheezing can be treated with drugs. Patients who have low blood oxygen concentrations are often given supplemental oxygen. Even though the overall prognosis of COPD patients has shown some improvement, the mortality rate remains high (Bruno and Valenti, 2012).

China has a long history of traditional medicines; the treatment of lung disease focuses the lung deficiency syndrome on purging the lung and dissolving phlegm. Many Chinese herbals have been used for several thousand years to treat respiratory diseases such as cough, asthma, bronchial ailments, and pneumonia (Jiangsu New Medical College, 1977; Lin, 2004; Wang and Ng, 2006; Zhou, 2005; Hsu et al., 2013).

In traditional Chinese medicine (TCM), *Pseudostellaria heterophylla* (TZS) can moisten the lung, cough, and is a spleen tonic. TZS is a mild herb that strengthens the Qi to tone up the body and resist pulmonary diseases (Yan, 2008). Some studies indicate that TZS possesses immunologic enhancement and antioxidant properties (Wong et al., 1994; Ng et al., 2004; Zhou, 2005; Huang et al., 2005). The ethyl acetate extract of TZS relieves cough and improves lung function *via* adjustment of the levels of multiple cytokines (Pang et al., 2011). The ethyl acetate extract of TZS contains cyclic-peptide compounds; the activity of these cyclic-peptide fractions is not clear.

In this paper, according to the clinical use of this herb in TCM (Murărescu et al., 2008), the present study was undertaken to evaluate cyclic-peptides separated from the ethyl acetate extract of TZS attenuating a COPD rat model induced by solid combustibles smoke (SCS), and also characterize the structures of the cyclic peptide monomer. Enzyme-linked immunosorbent assay (ELISA) was used to detect tumor necrosis factor (TNF-α) and interleukin-10 (IL-10), quantitative real-time polymerase chain reaction (q-PCR) and western blotting (WB) were used to detect the TLR4 mRNA, MyD88 mRNA and AP-1 (activator protein-1) mRNA meanwhile the downstream protein expression of p-p38 (phosphorylated protein 38), p-JNK (phosphorylated c-jun amino terminal kinase), IKK (inhibitor of nuclear factor κB kinas), p-IκB (phosphorylated inhibitor of NF-κB), and TAK1 (transforming growth factor beta-activated kinase 1) of the TLR4 pathway, regulated by CPE, to clarify its mechanism of action. Some particular molecules of cyclic-peptides are naturally present in *P. heterophylla* but their activity was seldom reported. It is of profound interest to elucidate this substance class.

## Materials and Methods

### Chinese TZS and Chemicals

Zheshen No. 2 P*. Heterophylla* (Miq.) Pax Chinese herbal was purchased from Ningde Nanling Agricultural Co., Ltd. Zherong County of the Ningde region of the Fujian Province in China is the advocate producing area of TZS. The TZS was cut into pieces and then dried below 60°C.

Chromatographic pure methanol was purchased from Merck KGaA (Darmstadt, Germany). Chromatographic pure acetonitrile was purchased from Fisher Scientific UK Ltd. (Leicestershire, England). Analytical grade reagents including petroleum ether, ethyl acetate, ethanol, n-butanol, ammonia, and chloroform were bought from Xilong Scientific Co., Ltd. (Shantou, China). Interleukin-10 (IL-10) and tumor necrosis factor (TNF-α) enzyme linked immunosorbent assay (ELISA) kits were purchased from cloud-clone corp.

### Instruments

A high performance liquid chromatography (HPLC) system with a 2996 photodiode-array detector (PDA) and an auto-sampler was used (Waters Technologies, USA). Liquid chromatography tandem mass spectrometry (LC-MS/MS) was performed with an Agilent 1290 ultra-high performance liquid chromatography (UPLC) combined with an AB Sciex Triple time of flight (TOF) 4600 MS system. A SpectraMax Plus384 multimode micro-plate reader (Molecular Devices, USA) and a Milli-Q-Plus ultra-pure water system (Millipore, Bedford, MA, USA) were used.

### CPE Preparation and Purification

The TZS was crushed to a powder (through 60 meshes) and was extracted using ethyl acetate as the solvent. According to the properties of the substance, the crude extract was purified by selecting the appropriate solvent to obtain the refined CPE.

**Figure 1:**
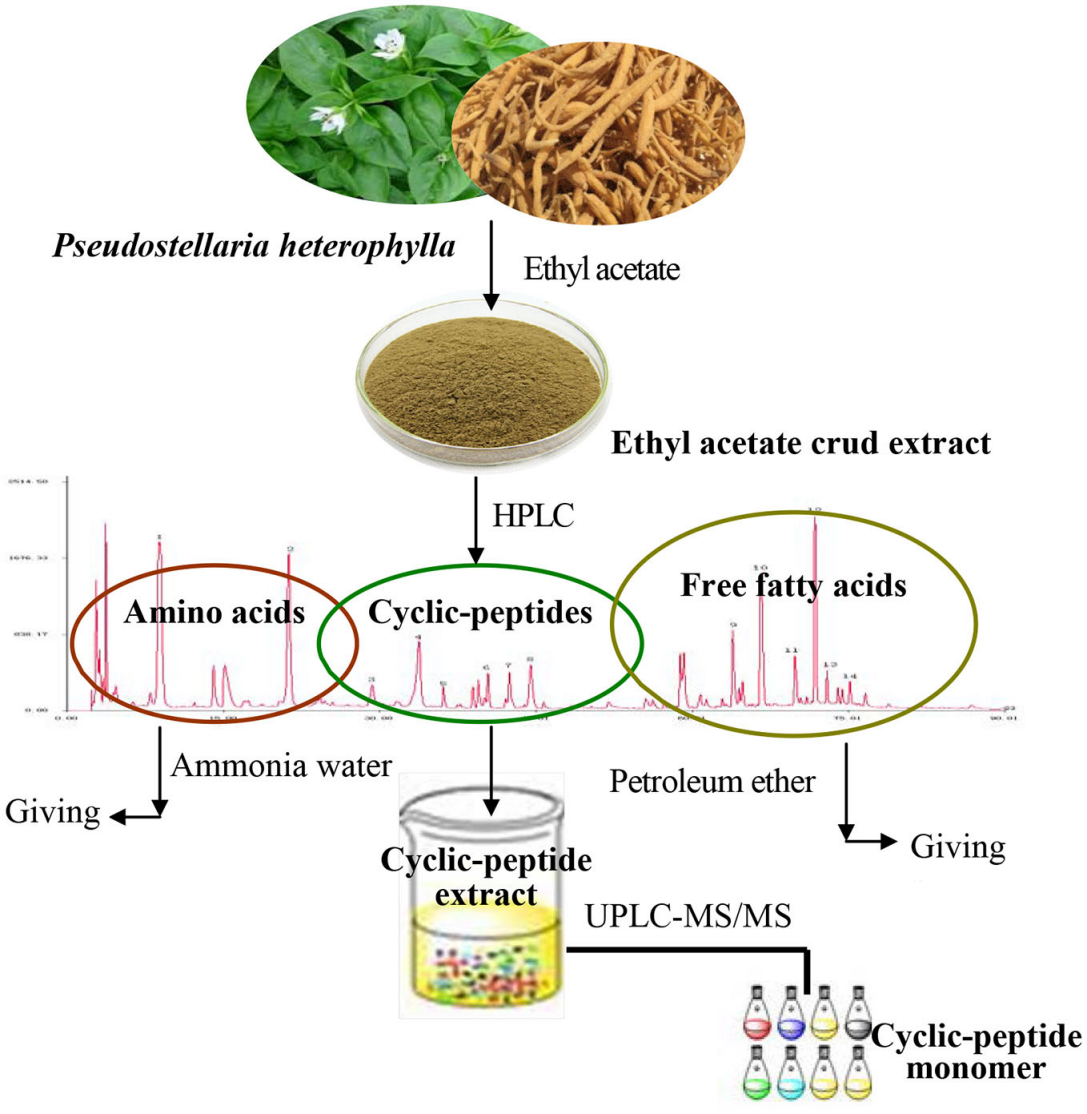
Diagrammatic sketch of cyclic-peptide extract preparation and purification.

Previous research of our group found that the ethyl acetate extract of TZS is rich in amino acids, cyclic peptides, and free fatty acids. Using several solvents to remove impurities, the process of CPE purification consisted of three steps: the ethyl acetate extract of TZS was degreased with petroleum ether, amino acids were removed with hot water, and polysaccharide and other impurities were removed with alcohol-precipitation. The purified cyclic-peptide powder was freeze-dried by lyophilization (**Figure 1**).

#### Ethyl Acetate Crude Extracts Preparation and HPLC-Fingerprint Analysis

TZS (60 mesh) was extracted with ethyl acetate using the Soxhlet extraction method for 2 h at 90°C. The extract was restored at room temperature, evaporated to dryness, and the ethyl acetate was removed in a rotary evaporator under reduced pressure. The residue was dissolved with chromatographic grade methanol, filtered through a 0.45 μm filter before analysis by HPLC. HPLC fingerprint analysis showed that the ethyl acetate extract contained three kinds of substances: amino acids, cyclic peptides, as well as free fatty acids. Thus, if pure cyclic peptides were to be obtained, the ethyl acetate extract needed to be further purified.

#### Removal of Fat-Soluble Substances

The ethyl acetate extract was degreased by mixing with petroleum ether (material: liquid = 1:30) to extract fat-soluble substances using ultrasonic extraction for 1 h at room temperature. The extract was filtered and the petroleum ether was discarded; the residue was degreased with petroleum ether in triplicate. The solvent was evaporated from the residue and ethanol was added (material: liquid = 1:30) for ultrasonic extraction at 60°C for 1 h. The extract was filtered, restored at room temperature, evaporated to dryness and alcohol was removed in a rotary evaporator under reduced pressure.

#### Removal of Amino Acids

The above-mentioned residue was fully dissolved in 50 ml hot water and extracted with ethyl acetate thrice (volume ratio 1:2) with a separating funnel; 100 ml of ethyl acetate was added each time. After the ethyl acetate phase was concentrated and dried, 50 ml hot water was added to the funnel to dissolve the residue, and 50 ml water-saturated n-butanol was added (volume ratio 1:1) and extracted thrice. The combined n-butanol extraction solution was poured into a separating funnel, 150 ml 40% ammonia test solution (volume ratio 1:1) was added to the n-butanol extraction, and the mixture was agitated well, and extracted thrice. The ammonia test solution extracts were discarded and the n-butanol phase was concentrated and dried. The residue was fully dissolved in 25 ml hot water, 50 ml chloroform was added (volume ratio 1:1) and extracted thrice. The chloroform phase was removed under reduced pressure, and the extract was concentrated and dried.

#### Removal of Polysaccharide and Other Impurities

The alcohol precipitation method was used to remove polysaccharides and other impurities. The above residue was placed in a beaker, dissolved with a proper amount of 85% ethanol, set for a period, filtered, and the resulting filtrate was a clear alcohol solution.

The above alcohol solution was placed in a beaker and mixed with distilled water (volume ratio 1:1) to obtain a 50% alcohol solution (freezing point −25°C). After pre-freezing at −80°C, a yellow powder was obtained by freeze-drying in a vacuum freeze-dryer.

#### Chemical Analysis of the Freeze-Dried Powder

**Figure 2:**
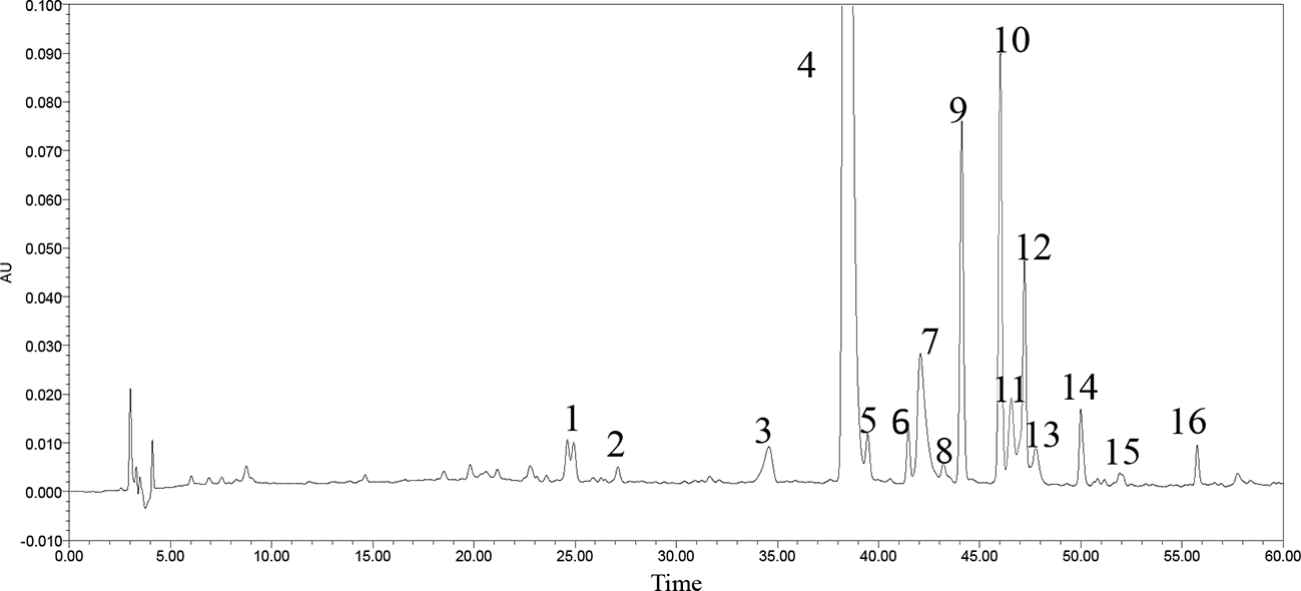
HPLC-fingerprint of cyclic-peptide extract.

A little powder was dissolved in methanol and filtered with a 0.22 μm microporous membrane. This paper deals with optimization of the chromatographic fingerprint of CPE to carry out HPLC analysis. The characteristic vibration cyclic-peptide bands showed the best responses at a wavelength of 203 nm, so 203 nm was successfully used to detect the target components. At the retention time of 25–60 min, 40 compounds with a peak area above 0.01% showed a better absorbance. For 16 of the 40 components, sixteen peaks were labeled with an area more than three ten thousand and the 40 components accounted for 96.9% of the total area (**Figure 2**).

### HPLC-Fingerprint Analysis of Extracts

The chromatography was carried out with a Hypersil ODS column (4.6 mm × 250 mm, 5 μm) made by Dalian Elite Analytical Instruments Co., Ltd. (Dalian, China). The mobile phase, flow rate and column temperature were optimized to obtain better resolution and establish an HPLC method to determine the fingerprint of the CPE. The mobile phase was acetonitrile (A) and 0.2% orthophosphoric acid aqueous solution (B).

The gradient elution program included five steps: From 0 to 40 min, the ratio of acetonitrile was increased from 10 to 40%; from 40 to 60 min, the ratio of acetonitrile was increased from 40 to 70%; from 60 to 75 min, the ratio of acetonitrile was increased from 70 to 100%; from 75 to 90 min, the acetonitrile was maintained at 100%. The detection wavelength was set at 203 nm; the flow rate was 1.0 ml/min, sample size was 20 μl, and the column temperature was 30°C.

### Cyclic-Peptide Ingredient Structure by LC-MS/MS Identification

UPLC-MS/MS determination was achieved on a SB C_18_ (2.1 × 100 mm, 1.8 μm) column. The velocity was 0.4 ml/min. The temperature of the auto-sampler and column were both maintained at 30°C. Both positive and negative electrospray ionization (ESI) modes were applied. The different instrument parameters including ion source temperature, collision energy, and collision energy spread were designed and optimized for high-quality fragment ions of the mass spectra (**Table 1**).

**Table 1:** CPE UPLC-MS/MS analysis conditions.

Chromatographic conditions		Mass spectrometry conditions	
Chromatographic column	SB C18 (2.1× 100 mm, 1.8 μm)	Polarity	Positive/Negative ion mode
Flow rate	0.4 ml/min	TOF mass range	Min 100, Max 1,500
Detective wavelength	203 nm	Ion Source	Gas 50, Curtain Gas 35
Sample size	1 μl	Ion Source Temp	500°C
Column temperature	30°C	Ion Spray Voltage Floating	5,020/−4,500
Mobile phase	0.1% glacial acetic acid aqueous solution (A)- acetonitrile (B)	Collision Energy (CE)	±40
Gradient program	0-11-12-17-19-20-23 min, 10-50-70-100- 100-10-10 B%	Collision Energy Spread (CES)	20

### COPD Rat Model Preparation and Treatment

SPF grade Wistar adult rats, male and female (50:50) weighing 200 ± 25 g were purchased from Shanghai Laboratory Animals Center (Shanghai, China) and fed in the animal center of our institution. The ambient temperature was 25°C and humidity 70%. Rats were allowed free access to tap water and were given a rodent diet.

Some 60 Wistar rats were randomly divided into six groups. The rats were exposed to the admixture of 40 g of cigarette and 300 g of wood chips. The exposure was performed in a 1 m^3^ fume cupboard with a flow rate of 0.1 m^3^/h and 60 min daily, for 60 days. Attention was paid to control the velocity of the flow of smoke, to properly ventilate, and to avoid accidental death of the animals. The control groups, consisting of 10 rats were unexposed to pollutants. Physiological saline was used as solvent to prepare suspension of CPE. Rats were orally given CPE at the low dose of 200 mg/kg (CPE-LD), medium dose of 400 mg/kg (CPE-MD), and high dose of 500 mg/kg (CPE-HD) for 15days.

Guilong Kechuanning capsule is a classical proprietary Chinese medicine (license number Z20053135). Guilong Kechuanning capsule is effective for lung diseases such as COPD and acute or chronic bronchitis, and for the improvement of immunological function and cellular cytokines such as IL-8 and TNF-α in patient with COPD (Huang and Cai, 1993; Li et al., 2005). In this study, rats were orally given physiological saline suspension of Guilong Kechuanning capsule (450 mg/kg/day) for 15 days that as a positive control.

The vehicle control and model control groups were given an equal amount of physiological saline by gastric perfusion, respectively.

### Pulmonary Function Assessment

Changes in the lung function of rats were observed using MedLab after treatment with CPE. Rats were anesthetized with pentobarbital sodium at a dose of 30 mg/kg and placed in a plethysmograph. Airway flow (V), tidal volume (V_t_), and trans-pulmonary pressure (Ptp) were monitored for 30 min and changes in each parameter were recorded. The effect of CPE was determined by comparing the COPD model changes in lung airway resistance (R_L_) and dynamic compliance (Cdyn) (Pang et al., 2011).

### Image Analysis of Lung Tissue Sections

Pulmonary tissue sections were made for pathological examination. A certain thickness of pulmonary tissue was made of random sections according to Valença et al. (Weibel and Gomez, 1962; Valença et al., 2004). Lung tissue sections were stained with hematoxylin and eosin (HE) and were studied morphologically under the light microscope. Dilated alveolar spaces and alveolar morphological characteristics were counted and identified using the 100× field of view for each group.

### Rat Serum Preparation, Alveolar Macrophage (AM) Isolation and Identification

When the CPE treatment was concluded, rats were given 1% pentobarbital sodium as an anesthetic. About 5 ml of blood was collected from inferior vena cava and was centrifuged for 20 min at 3,000 r/min to prepare serum was stored at −80°C for ELISA detection. The trachea and lungs were exposed and pulmonary alveolar macrophages (AMs) from each group were separated by bronchoalveolar lavage fluid (BALF) extraction. Briefly, a 2 mm diameter tube was inserted into the bronchus and 5 ml sterile saline was pumped in and then removed along with the fluid and cells; this was repeated four times. To amalgamate BALFs, the fluid was centrifuged for 15 min at 1,000 r/min. Cell precipitates were used to prepare single cell suspension by a floating and sinking process with RPMI-1640 medium containing 10% FBS, 100 U/ml penicillin, and 100 U/ml streptomycin. Giemsa stain was used to confirm AM purity >90% and trypan blue staining identified a cell survival rate ≥95%. AMs were inoculated in a culture dish at a concentration of 1 × 10^9^/ml under an atmosphere of 37°C, 5% CO_2_ for 2 h to gain the adherent cells. AMs were stored in liquid nitrogen for PCR and WB detection (Huang et al., 2015; Yu et al., 2018).

### Cytokine Measurement by ELISA

The levels of TNF-α and IL-10 in serum and the supernatant of AMs were detected by ELISA kits, respectively. The operation manual was read carefully before testing.

### Q-PCR Assay

RNA extraction of AMs was performed using a RNA Fast Mini kit (GK3016, Generay, Shanghai, China). According to the manufacturer’s instructions, RNA was extracted and stored at −80 °C. Reverse transcription was performed using the HiScript-II Q RT SuperMix for qPCR (Vazyme, Nanjing, China). A CFX connect Real-Time PCR System (Bio-Rad Laboratories, USA) was used to assay the gene expression of TLR4, MyD88, and AP-1 in AMs.

The PCR primers for TLR4, MyD88 and the internal reference, GAPDH, were designed based on DNA gene sequences from the GenBank website. The three pairs of primers were:

TLR4: F 5′- GACACTTTATCCAGAGCCGTTG -3′,
   R 5′- GGACTTCTCCACTTTCTCAAGG -3′
MyD88: F 5′- CAACCAGCAGAAACAGGAGTCT -3′,
   R 5′- ATTGGGGCAGTAGCAGATGAAG -3′
AP-1: 5′ -AAACGACCTTCTACGACGATG-3′,
   5′-TCGGAGGTGCGGCTTCAGATT-3′
GAPDH: F 5′- TATGACTCTACCCACGGCAAGT -3′,
   R 5′- ATACTCAGCACCAGCATCACC -3′

The PCR amplification reaction contained 10 µl 2× ChamQ SYBR Color qPCR Master Mix, 0.6 µl 10 µM forward primer, 0.6 µl 10 µM reverse primer, 2 µl template cDNA, and distilled water (dH_2_O) was added to 20 µl. PCR was conducted with the following reaction conditions: 95°C for 30 s, and 40 cycles of 95°C for 10 s, 59°C for 30 s, and 60 °C for 30 s. A melting curve was performed from 70 to 95 °C in increments of 0.5°C for 5 s.

### Western Blotting Analysis

The cells were harvested after treatment for 12, 24, and 48 h. AM lysate protein was extracted and analyzed by 12% sodium dodecyl sulfate polyacrylamide gel electrophoresis (SDS-PAGE) that was transferred to a polyvinylidene difluoride (PVDF) membrane, and then blocked for 1 h with 5% non-fat milk. The membrane was incubated overnight with the following primary antibodies: JNK, p38, IKK, IκB, TAK1, and AP-1 (1:1,000), and GAPDH (1:5,000). Membranes were immersed in 1× TBST buffer, washed on a shaker three times for 10 min, incubated with secondary antibodies (1:10,000) for 1 h, and the membranes were washed with TBST. The bands were quantified by image software.

## Results

### Freeze-Dried Powder Identification by UPLC-MS/MS Analysis

**Figure 3:**
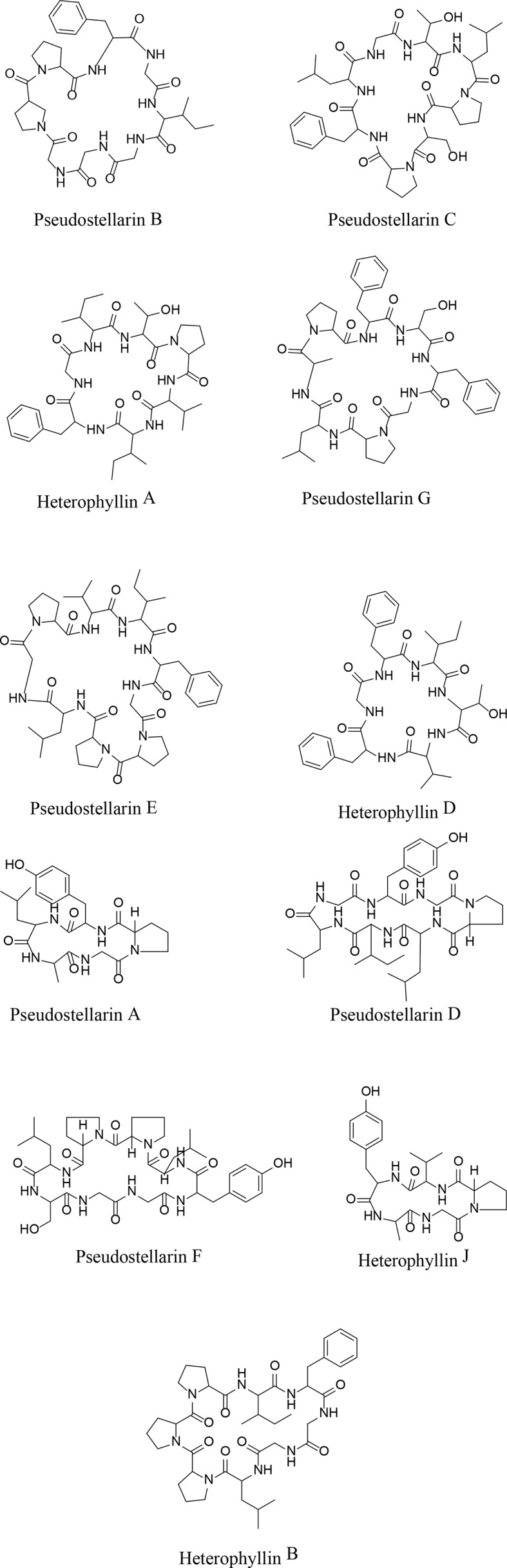
The structures of 11 kinds of cyclic peptide monomers.

Both positive and negative ESI modes were applied, and the positive ESI response was much better than the negative ESI response. ESI^+^ mode, (positive ions) such as [M + H]^+^ and [M + NH_4_]^+^, were measured in mass in a full scan. Molecular and characteristic fragment ions of 16 chemical components are seen in **Table 2**. Their structures were investigated through fragmentation reactions of mass spectrum and compared with the existing literature (Fu et al., 2012; Hua et al., 2017). [M + H]^+^ at *m/z* 432.2817 (M + NH4), *m/z* 488.2504, *m/z* 502.266, *m/z* 564.2817, *m/z* 683.3511, *m/z* 785.4192, *m/z* 779.445, *m/z* 813.4505, *m/z* 714.4185 *m/z* 728.4341, *m/z* 498.2459, *m/z* 817.4243, *m/z* 878.5135, *m/z* 665.3657, *m/z* 279.2319, *m/z* 277.2173(M–H), respectively. Peaks 1–16 was identified as Leu-Pro-Val-Ser molecules, Heterophyllin J, Pseudostellarin A, new Pseudostellarin, Pseudostellarin B, Pseudostellarin F, Heterophyllin B, Pseudostellarin C, Pseudostellarin D, Heterophyllin A, His-Gly-Trp-Val molecules, Pseudostellarin G, Pseudostellarin E, Heterophyllin D, octadecatrinoic acid, and linolenic acid/α-linolenic acid, respectively. There were 12 cyclic peptide monomer components of the 16 compounds; peak 4 was tentatively identified as one cyclic peptide compound and its structure must be further confirmed. The structures of 11 cyclic peptide monomers are shown in **Figure 3**. The relative contents in percentage were calculated using the area normalization method, after the ethyl acetate crude extract was purified to remove naturally occurring impurities, which indicated that the cyclic-peptides content in freeze-dried powder was up to 92.94%. Freeze-dried powder was the refined CPE.

**Table 2:** Peak area information of freeze-dried powder methanol solution.

HPLC-fingerprint analysis						UPLC-MS/MS analysis					
Marking peak no.	RT (min)	Peak area (μAμ)	Peak area percentage (%)	CP peak area percentage (%)	RT (min)	m/z(M + H)	Mass Error (ppm)	Fomula	MW.	Name	MS/MS
1	24.62	116,498	0.77	–	4.40	432.2817(M+NH4)	-5.9	C_19_H_34_N_4_O_6_	414.25	Leu Pro Val Ser	265.1616; 177.1112 133.0851; 89.0588
2	27.12	48,684	0.32	0.32	6.72	488.2504	-0.5	C_24_H_33_N_5_O_6_	487.24	Heterophyllin J	460.2562; 297.1904 169.1322; 70.0646
3	34.58	220,233	1.45	1.45	7.48	502.2660	-1	C_25_H_35_N_5_O_6_	501.26	Pseudostellarin A	474.2715; 361.1877 233.1282,70.0652
4	38.39	9,829,954	64.56	64.56	7.62	564.2817	-0.8	C_30_H_37_N_5_O_6_	563.27	new pseudostellarin	536.2865; 318.1449 155.0812,70.0650
5	39.46	141,146	0.93	0.93	8.62	683.3511	-0.2	C_33_H_46_N_8_O_8_	682.34	Pseudostellarin B	665.3406; 570.2670 212.1389
6	41.47	131,686	0.86	0.86	8.83	785.4192	-2.4	C_38_H_56_N_8_O_10_	784.41	Pseudostellarin F	757.4240; 672.3340 308.1957
7	42.07	741,190	4.87	4.87	9.45	779.445	-2.1	C_40_H_58_N_8_O_8_	778.44	Heterophyllin B	751.4508; 405.2494
8	43.20	37,451	0.25	0.25	10.02	813.4505	1.5	C_40_H_60_N_8_O_10_	812.44	Pseudostellarin C	795.4441; 471.2972 358.2137
9	44.11	912,328	5.99	5.99	10.09	714.4185	1	C_36_H_55_N_7_O_8_	713.41	Pseudostellarin D	686.4253; 601.3354 573.3397; 211.1442
10	46.01	1,049,294	6.89	6.89	10.61	728.4341	0.6	C_37_H_57_N_7_O_8_	727.43	Heterophyllin A	710.4245; 615.2519 310.2149; 197.1280
11	46.56	340,299	2.24	–	10.67	498.2459	-2.7	C_24_H_31_N_7_O_5_	497.24	His Gly Trp Val	212.1178; 110.0709
12	47.21	649,931	4.27	4.27	11.00	817.4243	-0.2	C_42_H_56_N_8_O_9_	816.42	Pseudostellarin G	799.4176; 555.2935; 467.2297; 332.1605
13	47.77	176,064	1.16	1.16	11.30	878.5135	0.6	C_45_H_67_N_9_O_9_	877.51	Pseudostellarin E	765.4313; 674.4264; 365.2195; 308.1971
14	49.99	211,700	1.39	1.39	12.10	665.3657	0.3	C_35_H_48_N_6_O_7_	664.36	Heterophyllin D	647.3575; 387.2019; 288.1325
15	51.92	57,081	0.37	–	16.13	279.2319	-0.2	C_18_H_30_O_2_	278.22	Octadecatrinoic acid	149.0231; 95.0852; 81.0700; 67.0546
16	55.75	88,005	0.58	–	19.93	277.2173(M-H)	0.3	C_18_H_30_O_2_	278.42	Linolenic acid	N/A
**Area proportion**			**96.9**	**92.94**							

### TZS Cyclic Peptide Anti-COPD Activity

#### The Safety Preliminarily Evaluating by Feeding Rats With CPE for 28 Days

According to the State Food and Drug Administration (SFDA) “Technical guidelines for repeated drug administration toxicity testing”, we tested the toxicity through twenty-eight days feeding study. A total of 100 SPF grade Wistar weaning rats, weighted (80 ± 10) g were divided randomly into five groups, 10 male rats and 10 female rats in each group, namely blank control group, vehicle control group, 200, 400 and 500 mg/kg/d dose of CPE group. Rats were fed the corresponding dose of drug or vehicle. On the 30th day, samples of blood were collected for hematological and biochemical analysis. Livers, spleens, kidneys and testicles (ovariesy) from all rats were weighed, furthermore, and the ratio of these organs to body weight was determined. The liver, kidneys, spleen, stomach, duodenum, testicles, ovariesy were removed for histopathological examinations. The tested animals were generally in good condition that both experimental groups and control group grown healthy and there was no abnormal change in body weight. Pathological examination of the experimental groups was negative. The results of hematological and biochemical indexes showed that all the indexes were in the normal range, the recommended dose of CPE was given to animals, and no toxicity was observed, results were listed **Tables 3A**, **B**.

**Table 3A:** Effects of CPE on hematological indexes in rats $\left(\bar{x}\pm sd\right)$.

Sex	Dose (mg/kg·bw/d)	n	Leucocyte count (×10^9^/L)	Red-cell count (×10^12^/L)	Hemoglobin (g/L)	Lymphocyte (%)	Neutrophil (%)	Monocyte (%)	Eosinophil (%)	Basicyte (%)
Female	Blank control	10	7.4 ± 1.9	7.3 ± 0.4	138 ± 4	85.0 ± 3.4	10.1 ± 3.9	3.12 ± 0.78	0.54 ± 0.19	0.19 ± 0.09
	Vehicle control	10	6.3 ± 1.8	7.4 ± 0.3	139 ± 5	84.1 ± 3.2	11.1 ± 2.8	3.30 ± 0.87	0.58 ± 0.18	0.13 ± 0.05
	200	10	7.5 ± 2.1	6.9 ± 0.6	132 ± 10	84.6 ± 3.6	10.9 ± 4.0	3.15 ± 0.71	0.42 ± 0.21	0.11 ± 0.07
	400	10	6.9 ± 1.7	7.2 ± 0.4	135 ± 8	85.0 ± 5.3	9.9 ± 4.2	3.37 ± 1.09	0.55 ± 0.20	0.13 ± 0.05
	500	10	6.0 ± 1.5	7.4 ± 0.3	140 ± 7	84.5 ± 2.6	10.3 ± 2.6	3.35 ± 0.84	0.68 ± 0.25	0.15 ± 0.07
Male	Blank control	10	6.8 ± 1.0	7.3 ± 0.3	138 ± 4	84.3 ± 2.8	11.7 ± 2.7	2.67 ± 0.49	0.54 ± 0.29	0.15 ± 0.07
	Vehicle control	10	6.3 ± 1.9	7.3 ± 0.4	137 ± 8	83.6 ± 3.3	12.4 ± 3.2	2.80 ± 0.91	0.52 ± 0.33	0.11 ± 0.07
	200	10	5.9 ± 1.5	7.5 ± 0.5	134 ± 6	82.6 ± 3.0	12.2 ± 2.7	2.72 ± 0.98	0.68 ± 0.39	0.13 ± 0.05
	400	10	7.0 ± 1.2	7.3 ± 0.2	138 ± 4	84.8 ± 3.3	11.0 ± 3.0	2.74 ± 0.93	0.58 ± 0.19	0.12 ± 0.04
	500	10	7.6 ± 1.2	7.3 ± 0.2	138 ± 3	83.8 ± 3.4	11.3 ± 3.1	3.27 ± 0.79	0.62 ± 0.17	0.18 ± 0.04

**Table 3B:** Effects of CPE on blood biochemical indexes in rats $\left(\bar{x}\pm sd\right)$.

Sex	Dose (mg/kg·bw/d)	n	Alanine transaminase (U/L)	Aspertate Aminotransferase (U/L)	Total protein (g/L)	Albumin (g/L)	Blood urea nitrogen (mmol/L)	Creatinine (μmol/L)	Total cholesterol (mmol/L)	Triglyceride (mmol/L)
Female	Blank control	10	32 ± 2	115 ± 18	54.2 ± 1.9	32.9 ± 1.9	5.84 ± 0.91	33 ± 5	1.88 ± 0.32	0.40 ± 0.09
	Vehicle control	10	34 ± 6	112 ± 20	53.3 ± 2.7	32.7 ± 1.5	5.79 ± 0.85	33 ± 4	1.93 ± 0.27	0.37 ± 0.17
	200	10	31 ± 5	95 ± 19	54.2 ± 3.0	33.1 ± 1.3	5.86 ± 1.03	36 ± 6	1.96 ± 0.29	0.31 ± 0.05
	400	10	30 ± 3	102 ± 10	55.1 ± 1.5	33.4 ± 1.0	5.47 ± 1.08	32 ± 6	1.98 ± 0.33	0.39 ± 0.09
	500	10	32 ± 4	112 ± 15	54.4 ± 2.3	33.1 ± 1.1	5.86 ± 0.84	33 ± 6	2.04 ± 0.35	0.33 ± 0.07
Male	Blank control	10	39 ± 4	129 ± 32	53.2 ± 1.2	31.9 ± 0.6	4.65 ± 0.59	24 ± 2	1.39 ± 0.28	0.48 ± 0.06
	Vehicle control	10	41 ± 8	142 ± 34	54.1 ± 1.6	32.3 ± 0.7	5.21 ± 0.82	28 ± 9	1.46 ± 0.29	0.43 ± 0.27
	200	10	44 ± 2	159 ± 41	52.7 ± 1.9	31.6 ± 0.8	5.86 ± 1.03	36 ± 6	1.96 ± 0.29	0.31 ± 0.05
	400	10	37 ± 4	127 ± 20	54.3 ± 1.6	32.3 ± 0.6	4.84 ± 0.52	25 ± 3	1.36 ± 0.30	0.47 ± 0.08
	500	10	38 ± 7	134 ± 21	54.5 ± 1.8	32.4 ± 1.0	4.83 ± 0.68	26 ± 2	1.40 ± 0.35	0.45 ± 0.07

#### CPE Improves Pulmonary Function

**Figure 4:**
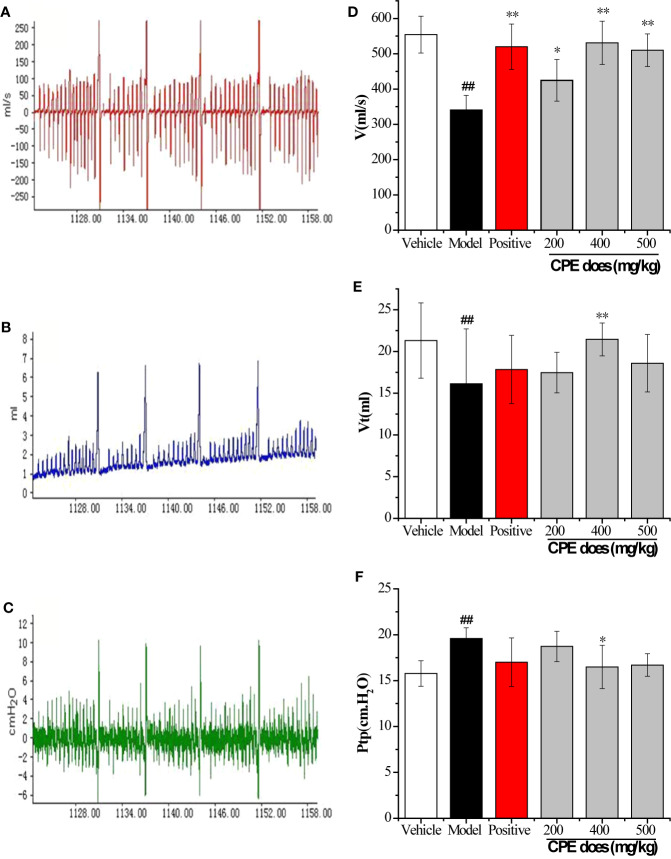
Results of pulmonary function parameters test. **(A)** The test waveforms of airway flow, **(B)** tidal volume, and **(C)** trans-pulmonary pressure; **(D)** the test results in airway flow, **(E)** tidal volume, and **(F)** trans-pulmonary pressure; The measurements were used to calculate R_L_ and Cdyn. *p < 0.05, **p < 0.01, compared with COPD model; ^#^p < 0.05, ^##^p < 0.01, compared with vehicle control, ($\bar{x}\pm sd$, *n* = 10).

Pulmonary ventilation function is an important index in COPD diagnosis and treatment. Results are increased lung airway resistance (R_L_) but decreased dynamic compliance (Cdyn). R_L_ and Cdyn are commonly measured by relating airflow and driving pressure. These data were calculated by measuring airway flow (V), tidal volume (V_t_), and trans-pulmonary pressure (Ptp) by the formula R_L_ = Ptp/V, Cdyn = V_t/_Ptp. The breathing rate of rats was recorded by the MedLab system for 30 min and the test waveforms are shown in **Figures 4A–C**. Compared with the vehicle control group, *V* and *V*_t_ were decreased in the COPD model rat, but led to a rise in Ptp. CPE (200, 400, or 500 mg/kg/day) was given orally for 15 days and the results showed that 400 mg/kg/day dose was the best dose. The V, and Vt were markedly increased (*p <*0.01) and Ptp was effectively lower (*p <*0.05) after treatment (**Figures 4D–F**). Compared with the vehicle control group, model group rats R_L_ increased by 67.12% (*p <*0.01) and Cdyn decreased by 75.14% (*p <*0.01). In the Guilong Kechuanning capsule positive group compared with the model group, R_L_ decreased by 26.02% and Cdyn increased by 140.40% (*p <*0.01). In the 400 mg/kg dose of CPE group compared with the model group, R_L_ decreased by 36.29% and Cdyn increased by 171.30% (*p <*0.01). Experimental results showed that CPE could decrease airway resistance and increased dynamic compliance in rats with COPD (**Table 4**).

**Table 4:** Changes of airway resistance and dynamic compliance in rats.

Groups	V (ml/s)	Vt (ml)	Ptp (cm·H2O)	R_L_ (cm·H_2_O·s/ml)	Cdyn (ml/cm·H_2_O)
Vehicle	554.23 ± 53.96	21.31 ± 4.42	15.77 ± 1.39	0.028 ± 0.001	1.351 ± 0.459
Model	340.82 ± 39.30^▲▲^	16.13 ± 6.59^▲▲^	19.60 ± 1.05^▲▲^	0.057 ± 0.005^▲▲^	0.823 ± 0.269^▲▲^
Positive	519.85 ± 63.82**	17.84 ± 4.06	16.70 ± 3.01*	0.033 ± 0.007**	1.050 ± 0.279*
CPE-LD	514.56 ± 73.71**	17.46 ± 2.36	18.72 ± 1.74	0.036 ± 0.006*	0.933 ± 0.422*
CPE-MD	530.96 ± 60.12**	21.45 ± 2.00**	16.49 ± 2.37*	0.031 ± 0.005**	1.301 ± 0.337**
CPE-HD	509.98 ± 44.23**	18.59 ± 3.41*	16.70 ± 1.21*	0.033 ± 0.008**	1.113 ± 0.471*

Values are expressed as $\bar{x}\pm sd$, n = 10.

^▲^P ≤0.05 vs vehicle control, ^▲▲^P ≤0.01 vs vehicle control.

*P ≤0.05 vs COPD model, **P ≤0.01 vs COPD model.

#### Image Analysis of Lung Tissue Sections

**Figure 5:**
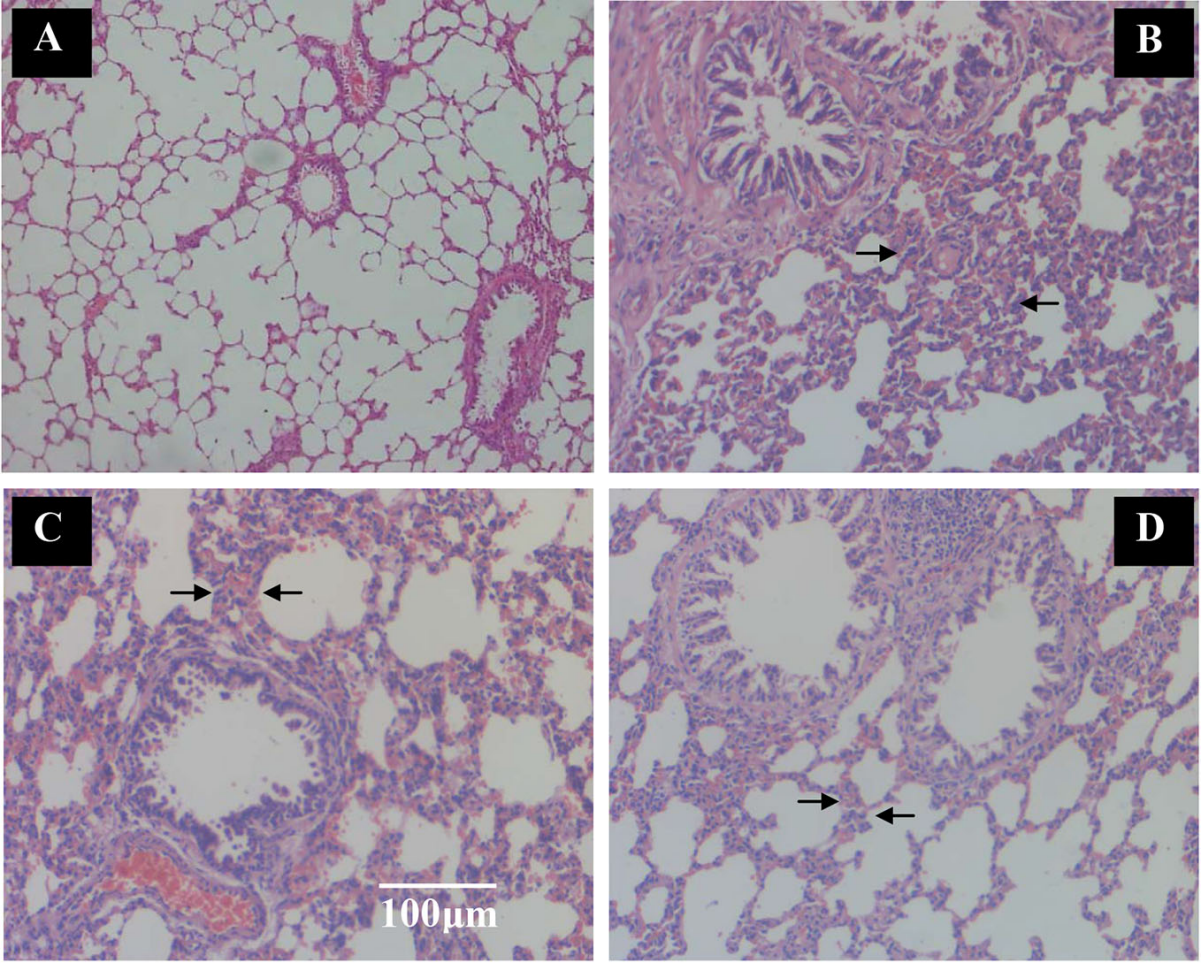
The effect of TZS extracts on alveoli and inflammatory cell infiltration in lungs in SCS-induced rats (hematoxylin and eosin stain). Vehicle control group and model group rats treated with saline, positive control group rats treated with Guilong Kechuanning capsule (450mg/kg/day) and drug group treated with CPE (400 mg/kg/day, orally) was given to SCS-induced rats, tissue histological examination were assessed. **(A)** Vehicle control group, **(B)** SCS model group, **(C)** positive control group and **(D)** CPE treatment group. Arrows indicate alveoli space and inflammatory cell infiltration. With CPE medication, alleviate the lung inflammation, more alveolar space and the infiltrations of inflammatory cells were improved, magnification ×100.

Rats were killed to obtain lung tissues when the CPE treatment concluded and the pulmonary tissue pathology was observed with HE staining. Crucial gas exchange takes place in the alveoli. There were full alveoli, respiratory ducts, peripheral, small airways that were normally present in the pulmonary parenchyma in the control group (**Figure 5A**). The number of alveoli was significantly decreased in the SCS group; rats with COPD had significant inflammation that was caused by inflammatory cell infiltration and damage to alveoli structure. The lung lesions were characterized by interstitial edema, the alveolar wall was thickened and there was less alveolar space. There was extensive necrotic changes of the alveoli and partial consolidation, and infiltration of inflammatory cells (**Figure 5B**). The lung damage treated with Guilong Kechuanning capsule (450 mg/kg/day) and CPE (400 mg/kg/day, orally) was lower than that in the model group. Image analysis suggested that CPE could decrease the degree of alveolar destruction (*p* < 0.05), alleviate lung inflammation, increase alveolar space, and improve infiltration of inflammatory cells in airway inflammation (**Figures 5C, D**).

### Effect of CPE on the Expression of Toll-Like Receptors and the Downstream Signaling Transduction Pathway in Rat AMs

#### Effect of CPE on the Proliferation of Rats Alveolar Macrophage

CPE of different concentrations was used to treat the cells at the 24, 48 and 72 h, and cell proliferation was observed using MTT method to evaluate the cytotoxicity of CPE to AMs. AMs proliferation rates were 100% 72 h at the CPE of 50 μg/ml, 99.73% 72 h at the concentration of 100 μg/ml (P <0.05), 99.55% 72 h at the concentration of 200 μg/ml (P <0.05), 99.28% 72 h at the concentration of 500 μg/ml (P <0.05), 95.83% 72 h at the concentration of 1,000 μg/ml (P <0.05), and 88.08% 72 h at the concentration of 2,000 μg/ml (P <0.05). When the concentration of CPE was lower than 1000 μg/mL, the CPE showed no toxicity on AMs.

#### Cytokine Measurement by ELISA in Serum and Cell Supernatants

Medication group rats were treated with intragastric administration of CPE (200, 400, and 500 mg/kg) once daily for 15 days. Orbital plexus blood samples were collected; serum was isolated and assayed by ELISA. AMs of each group were separated by bronchoalveolar lavage.

**Figure 6:**
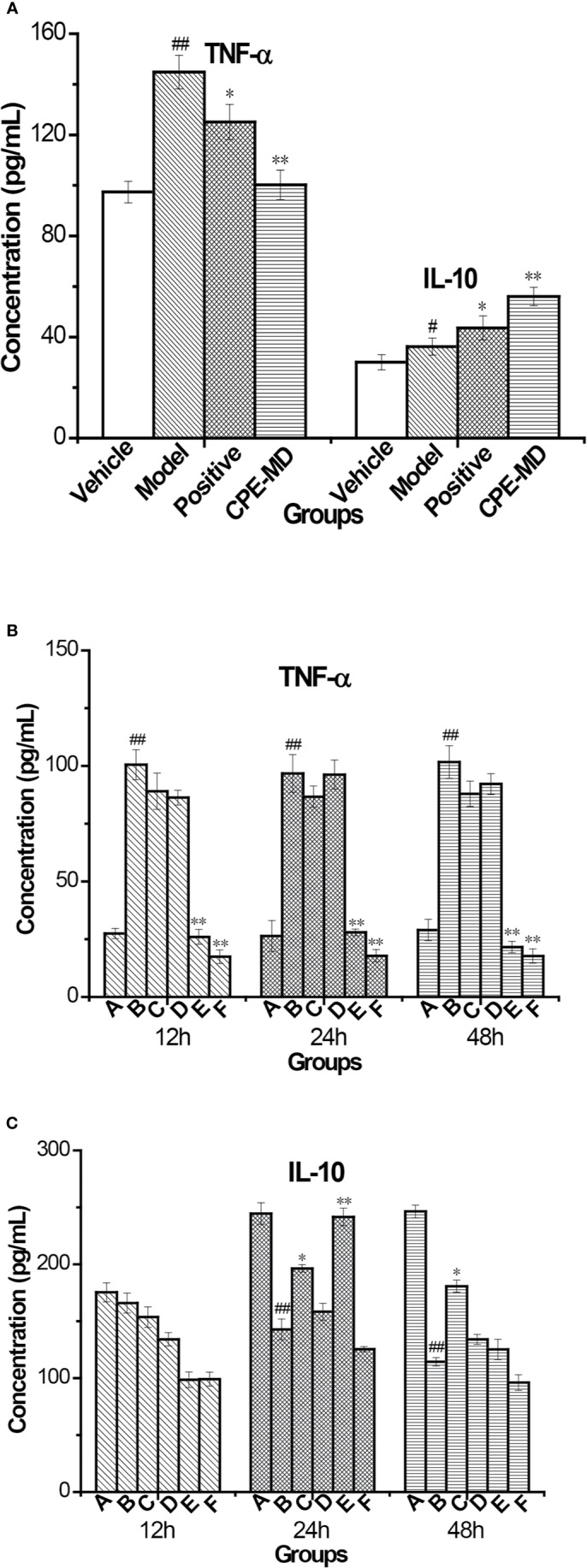
Changes of cytokine in rat serum and supernatant of AMs with COPD model treatment with CPE. **(A)** TNF-α and IL-10 level in rat serum ($\bar{x}\pm sd$, *n* = 10). **(B)** TNF-α level and **(C)** IL-10 level in supernatant of AMs. *p < 0.05, **p < 0.01, compared with COPD model; ^#^p < 0.05, ^##^p < 0.01, compared with vehicle control; **A—**Vehicle control, **B—**COPD model + LPS, **C—**positive + LPS, **D—**CPE-LD + LPS, **E—**CPE-MD + LPS, **F**—CPE-HD + LPS; *p < 0.05, **p < 0.01, compared with COPD model; ^#^p < 0.05, ^##^p < 0.01, compared with vehicle control, ($\bar{x}\pm sd$, *n* = 6).

TNF-α and IL-10 cytokine in the serum were measured using ELISA. COPD model group, the level of TNF-α inflammatory cytokine was increased in the serum when compared to the vehicle control group (145.34 ± 16.92 pg/ml *vs* 97.72 ± 6.94 pg/ml, *p <*0.01). Treatment with CPE resulted in significant differences (*p <*0.01) in that the concentration of TNF-α dropped from 145.34 ± 16.92 to 100.47 ± 8.01 pg/ml. However, the concentration of IL-10 anti-inflammatory cytokine was also increased in the serum of model rats (36.57 ± 1.63 pg/ml *vs* 29.80 ± 0.85 pg/ml, *p <*0.01). Guilong Kechuanning capsule and CPE groups, the levels of serum IL-10 were markedly increased to 43.13 ± 2.70 and 55.72 ± 2.67 pg/ml, respectively (*p <*0.01). For treatment with the positive control and CPE, levels of TNF-α decreased, but IL-10 increased, suggesting that they have obvious anti-inflammatory effects. A CPE dose of 400 mg/kg was the best and its curative effect was better than the positive control (**Figure 6A**).

After treatment of CPE, pulmonary alveolar macrophages (AMs) of all experiment group rats were separated by bronchoalveolar lavage fluid extraction. AMs were stimulated with lipopolysaccharide (LPS) (end concentration 0.1 μg/ml) and cultured with RPMI-1640 containing 10% FBS medium for different time (12, 24, or 48 h), AMs separated by normal and model groups rat served as controls. The levels of TNF-α and IL-10 in the AMs supernatant were detected by ELISA. The level of TNF-α was decreased treatment with CPE, but increased level of IL-10 in supernatant. AMs isolated from rats were treated with 400 mg/kg of CPE (CPE-MD) and cultured for 24 h that it was the best results. The concentration of TNF-α and IL-10 in the supernatant of AMs (vehicle or model group) was 26.37 ± 2.10, 96.79 ± 3.18, 244.59 ± 8.43, and 142.77 ± 6.28 pg/ml. CPE-MD group, they were 27.94 ± 2.15 and 241.57 ± 7.20 pg/ml; respectively. More detailed data are illustrated in **Figures 6B, C**.

#### Expression of TLR4 mRNA and MyD88 mRNA of AMs by RT-PCR

An AM cell line was stimulated with LPS and cultured with RPMI 1640 containing 10% FBS medium. Twenty-four hours later, RNA of AMs was extracted and TLR4 mRNA MyD88 mRNA, and AP-1 mRNA levels were determined with relative quantification using the 2^−△△Ct^ method.

TLR4 mRNA expression in the COPD model group was 2.98 ± 0.13, which was remarkably increased compared to the vehicle control group (1.00 ± 0.00, *p <*0.01). CPE could attenuate the expression of TLR4 mRNA in COPD rats. TLR4 mRNA levels were 2.09 ± 0.18 for the CPE-LD group, 1.10 ± 0.10 for the CPE-MD group, and 1.09 ± 0.22 for the CPE-HD group. TLR4 mRNA expression was decreased (*p <*0.01) after using CPE-MD or CPE-HD, while it increased in the CPE-LD group (*p <*0.05).

MyD88 mRNA expression in AMs of the COPD rats (3.08 ± 0.22) was increased compared to that of vehicle groups (1.26 ± 0.28). In the CPE-LD group it was 2.58 ± 0.26, 1.33 ± 0.16 in the CPE-MD group, and 1.57 ± 0.10 in the CPE-HD group. The MyD88 mRNA expression was decreased (*p <*0.01) after using CPE-MD or CPE-HD, while it increased in the CPE-LD group (*p <*0.05).

AP-1 mRNA expression in AMs of the COPD rats (1.22 ± 0.05) was increased compared to that of vehicle groups (1.00 ± 0.00). In the CPE-LD group it was 1.13 ± 0.05, 1.08 ± 0.11 in the CPE-MD group, and 1.09 ± 0.08 in the CPE-HD group. The AP-1 mRNA expression was decreased (*p <*0.05) after using CPE-MD or CPE-HD, but not significantly difference with CPE-LD group.

CPE reduced the mRNA level of TLR4, MyD88, and AP-1 exerted anti-inflammatory effects, and simultaneously resistance LPS infection to stimulus. Vehicle control values of TLR4, MyD88 and AP-1 were expressed as 1.0. Results were listed in **Table 5**.

**Table 5:** Effect of CPE on the expression of TLR 4 mRNA, MyD88 and AP-1 mRNA in AMs.

Groups	2^−△△ct^(TLR4)	2^−△△ct^ (MyD88)	2^−△△ct^ (AP-1)
Vehicle	1.00 ± 0.00	1.00 ± 0.00	1.00 ± 0.00
Model	2.98 ± 0.13^▲▲^	2.44 ± 0.17^▲▲^	1.22 ± 0.05^▲^
Positive	1.74 ± 0.19**	1.41 ± 0.12**	1.19 ± 0.07
CPE-LD	2.09 ± 0.18*	2.05 ± 0.21*	1.13 ± 0.05
CPE-MD	1.10 ± 0.10**	1.06 ± 0.13**	1.08 ± 0.11*
CPE-HD	1.09 ± 0.22**	1.25 ± 0.08**	1.09 ± 0.08*

Values are expressed as $\bar{x}\pm sd$, n = 6.

^▲^P ≤0.05 vs vehicle control, ^▲▲^P ≤0.01 vs vehicle control.

*P ≤0.05 vs COPD model, **P ≤0.01 vs COPD model.

#### Levels of p-p38, p-JNK, p-IKK, p-IκB, p-TAK1, and AP-1 in AMs by WB

An AM cell line was stimulated with LPS and cultured with RPMI 1640 containing 10% FBS medium. Twenty-four hours later, total protein was extracted. Western blot was used to determine the total protein levels of p38, JNK, IKK and levels of phosphorylated IKK, JNK, and p38 in whole-cell lysates. The expression of p-IκB protein was unstable in AMs. Compared with the vehicle control group, the protein levels of p-p38, p-JNK, p-IKK, p-TAK1, and AP-1 were significantly increased in the COPD model group while the expression of p-IKK in the CPE group was not significantly decreased compared to the model group (*p >*0.05). p-JNK proteins in the CPE-LD, CPE-MD, and CPE-HD groups were significantly decreased compared with the COPD model group (*p <*0.01) and p-p38 proteins in the CPE-MD, and CPE-HD groups were significantly decreased compared with the COPD model group (*p <*0.01). AP-1 protein in the CPE-MD group was decreased compared with the COPD model group (*p <*0.05). p-TAK1 proteins in the CPE-MD and CPE-HD groups were significantly decreased compared with the COPD model group (*p <*0.01).

**Figure 7:**
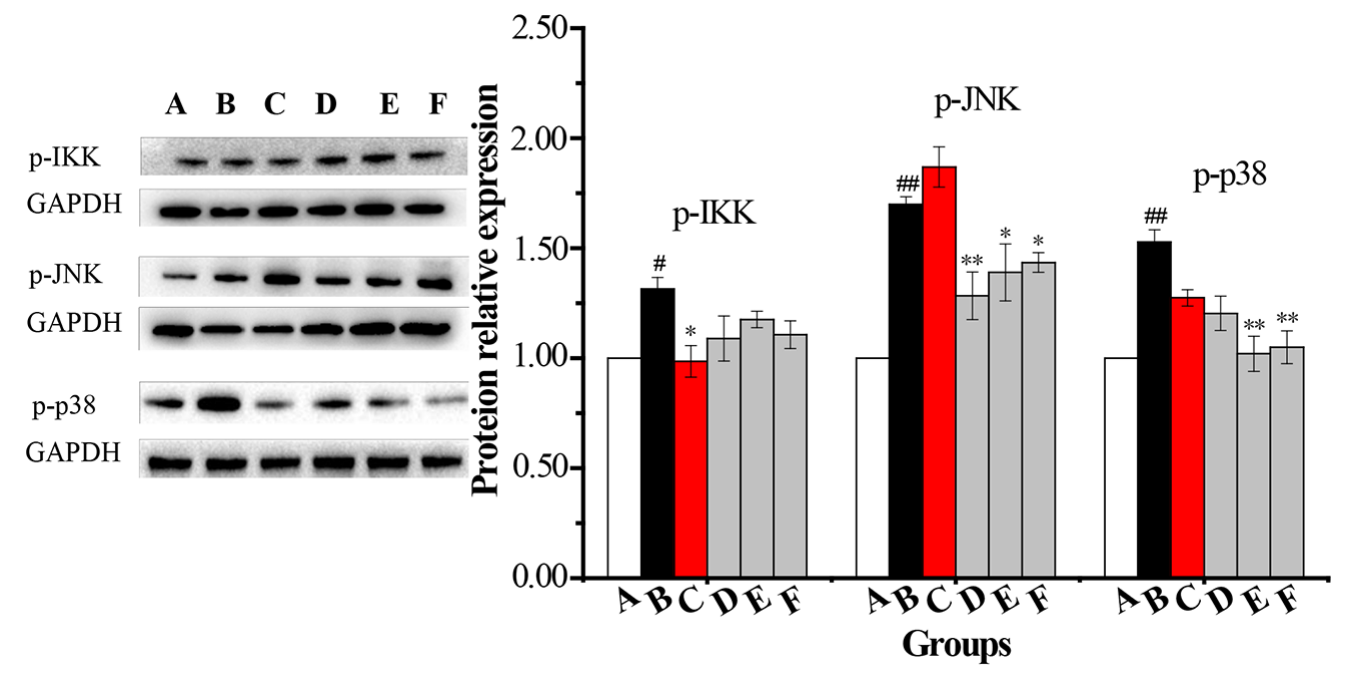
The changes in expression levels of p-IKK, p-JNK and p-p38 in rat alveolar macrophages after the treatment with CPE. **A—**vehicle control, **B—**COPD model + LPS, **C—**positive + LPS, **D—**CPE-LD + LPS, **E—**CPE-MD + LPS, **F**—CPE-HD + LPS; *p < 0.05, **p < 0.01, compared with COPD model; ^#^p < 0.05, ^##^p < 0.01, compared with vehicle control, ($\bar{x}\pm sd$, *n* = 6).

**Figure 8:**
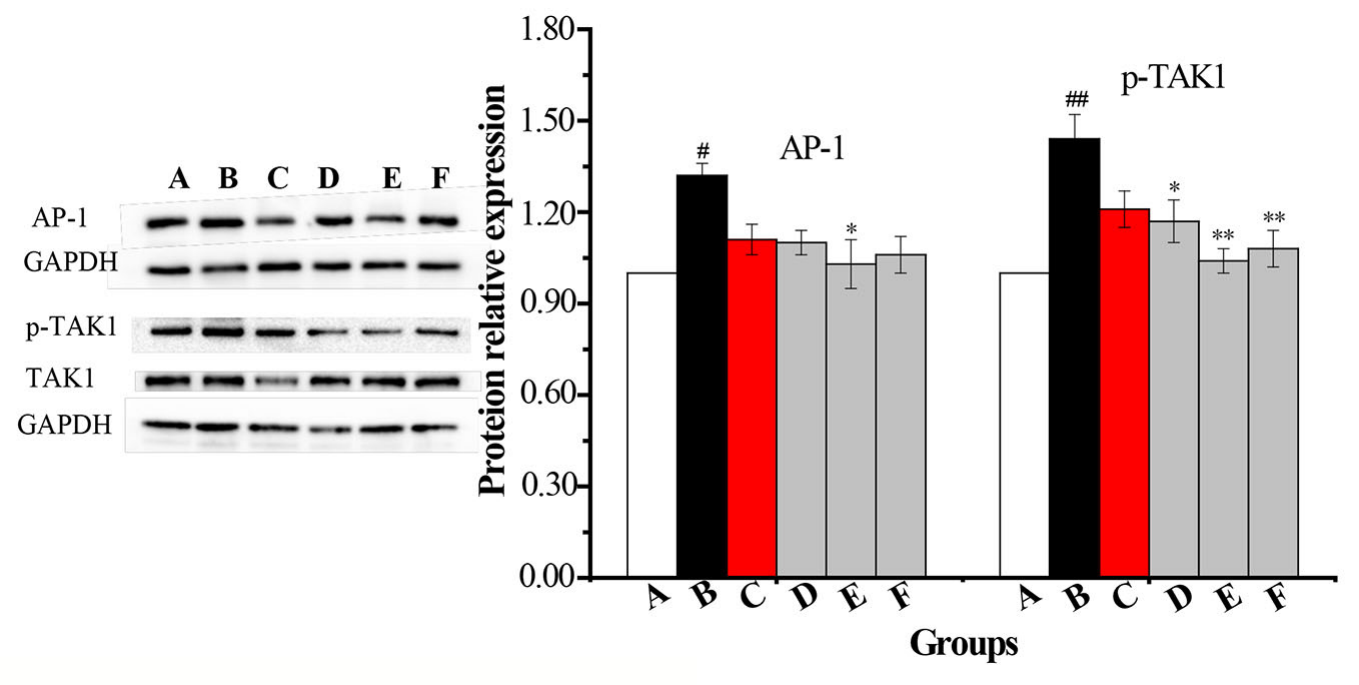
The changes in expression levels of AP-1 and p-TAK1 in rat alveolar macrophages after the treatment with CPE. **A—**vehicle control, **B—**COPD model + LPS, **C—**positive + LPS, **D—**CPE-LD + LPS, **E—**CPE-MD + LPS, **F**—CPE-HD + LPS; *p < 0.05, **p < 0.01, compared with COPD model; ^#^p < 0.05, ^##^p < 0.01, compared with vehicle control, ($\bar{x}\pm sd$, *n* = 6).

CPE reduced the proteins level of p-JNK, p-p38, p-TAK1, and AP-1 exerted anti-inflammatory effects, and simultaneously resistance LPS infection to stimulus. The levels of p-IKK, p-JNK, p-p38, p-TAK1, and AP-1 have been normalized by the levels of the corresponding total protein and expressed relative to the phosphorylation ratio in vehicle-treated cells (see in **Figures 7** and **8**).

## Discussion

COPD is a public health problem. It exhibits symptoms of chronic bronchitis and emphysema, which causes the characteristic narrow airways and shortness of breath in patients. COPD drug therapy, including bronchodilators, anti-inflammatory agent, antioxidant, protease inhibitors, antibiotics, etc; but has a poor prognosis, at present there is no specific way to make COPD are completely curable. The primary aim of COPD treatment is to control symptoms, reduce the rate of deterioration and mortality, and improve the quality of life of patients (Fujii et al., 1999; Puljic et al., 2007; Wollin and Pieper, 2010; Zeng et al., 2010).

TNF-α is a pro-inflammatory cytokine that is closely correlated with pulmonary inflammation caused by COPD. IL-10 decreases pulmonary neutrophilia and also suppresses the expression of TNF-α, IL-6, and IL-8 (Pelaia et al., 2006). TLR4 mediates pathogen-induced NF-κB activation in endothelial cells through the homologous structure of the intracellular interleukin-1 receptor (IL-1R) followed by downstream signaling of MyD88, which then secretes immune inflammatory cytokines, and thereby causes COPD. With better understanding of the COPD inflammatory mechanisms, broad spectrum anti-inflammatory drugs may be more effective, such as inhibitors of PDE4, p38 MAPK and NF-κB, some data suggest that anti-inflammatory therapies might provide a way to prevent COPD condition worsens (Barnes, 2008).

Natural medicinal or Chinese medicine displays a unique chemical diversity and diversity of the biological activities which is one of the most important resources of novel lead compounds especially for the critical diseases. The research on active constituent of natural or traditional medicine is known as one of important means of developing new drugs.

TZS is a Chinese herb that resists pulmonary diseases. We previously showed that the ethyl acetate extract of TZS relieves cough and improves lung function *via* adjustment of the levels of multiple cytokines (Pang et al., 2011). The ethyl acetate extract of TZS is rich in cyclic-peptides; the activity of these cyclic-peptide fractions is not clear.

Cyclic peptides are the substances which are Head-totail cyclization refers to the formation of an amide bond between the C-terminal carboxylic acid and N-terminal amine of the peptide. Cyclic peptides possess diverse biological activities such as antibacterial, anticancer, antifungal, antiviral, and anti-inflammatory properties. More than 40 cyclic peptide drugs have been clinically approved, the most of which are derived from natural world. But some cyclic peptides there are problems with the metabolic stability and oral absorption limits their development as drug candidates (Jing and Jin, 2020).

In this paper, the ethyl acetate extract of TZS was purified that degreased with petroleum ether, amino acids were removed with hot water, and polysaccharide and other impurities were removed with alcohol-precipitation. The purity of CPE after purification is up to 92.94%.

Our research provides further evidence for *P. heterophylla* (Miq.) Pax. CPE prevents alveolar destruction and alleviating lung inflammation in COPD rats induced by SCS. The release of the pro-inflammatory cytokine TNF-α was significantly inhibited, while the anti-inflammatory cytokine IL-10 was increased in serum. These results are closely associated with airflow limitation in the COPD rat.

TLR4 and MyD88 expression was determined using SYBR Green I by real-time PCR. AMs were stimulated by LPS, the expressions of TLR4, MyD88 and p-JNK and p-p38 were enhanced, showing remarkably higher in COPD groups than normal control groups. Upon activation by LPS, TLR4 can form a dimer to recruit MyD88 and/or TRIF, and then bind with IRAK4 to enable IRAK1 to recruit TRAF6. The IRAK1–TRAF6 complex phosphorylates TAB2/TAB3 and TAK1 and thus activates the IKK and MAPK signaling pathways. JNK and p38 MAPK activation initiate the transcriptional potential of c-Jun or c-fos, a critical part of AP-1. After medication, the expression levels of TLR4 mRNA and MyD88 mRNA, and p-JNK and p-p38 were significantly down-regulated. Our experiments have proved that CPE inhibited TAK1 phosphorylation and effectively down-regulating the expression of AP-1. CPE intervention could improve pathological changes of the pulmonary ventilation function in COPD rats, which may be related to its effect in inhibiting abnormal activation of the TLR4-MyD88-JNK/p38 signal transduction pathway.

## Conclusion

In conclusion, we studied the CPE of TZS that it prevented alveolar destruction and alleviated lung airway inflammation by inhibiting inflammatory cytokine levels *via* the regulation of the TLR4/MyD88 pathway and its related proteins in COPD rats. This is the first report on cyclic peptides extracted from *P. heterophylla* lessens the severity of COPD episodes. The results of studies will be helpful to develop potential anti-COPD cyclic peptide drugs and solve tricky medical problems by stealing an idea from nature.

## Data Availability Statement

The datasets generated for this study are available on request to the corresponding authors.

## Ethics Statement

The animal study was reviewed and approved by Ethics committee on experimental animals of Fujian Academy of Traditional Chinese Medicine No. FJATCM-IAEC2017012.

## Author Contributions

FL: research. HY: separation experiment. S-DL: HPLC analysis and pharmacological experiments. LZ: UPLC/MS/MS analysis and pharmacological experiments. CJ: HPLC analysis and pharmacological experiments. Z-BC: statistical analysis. Y-YL: cell experiment and q-PCR assay. Y-JK: The safety evaluating and western blotting analysis. JH: project management and thesis writing. W-SP: CPE preparation methodology research.

## Funding

The authors gave thanks to Project 81673575, supported by the National Natural Science Foundation of China; the authors gave thanks to Project 2019YFC1710504, supported by the National Key R & D Plans.

## Conflict of Interest

The authors declare that the research was conducted in the absence of any commercial or financial relationships that could be construed as a potential conflict of interest.
